# Supplementary material for: Exonuclease action of replicative polymerase gamma drives damage-induced mitochondrial DNA clearance
Source: EMBO Rep. 2025 Jan 31;26(5):1385–405. doi: 10.1038/s44319-025-00380-1 (PMC11894172; doi:10.1038/s44319-025-00380-1)
Supplement: Supplementary file 9 — Expanded View Figures [file 44319_2025_380_MOESM9_ESM.pdf]

Expanded View Figures

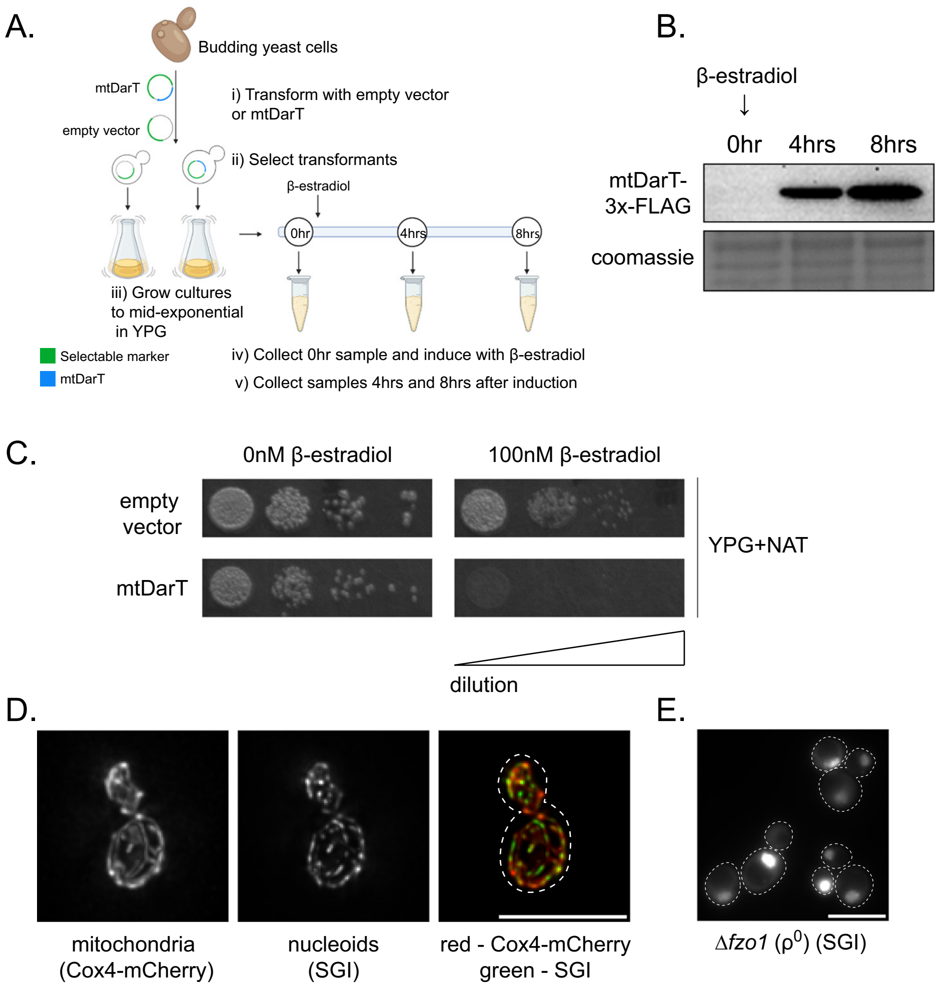

Figure EV1. mtDNA damage results in mtDNA clearance.

(**A**) Experimental setup followed for inducing mtDNA damage using the mtDarT system is shown (Dua et al, 2022). Cells were grown in a non-fermentable carbon source (YPG) and cultures were maintained in mid-exponential growth during the course of the entire experiment. OD600 at the time of imaging was always ensured to be comparable between control (empty vector) and mtDarT-treated cells. Schematic created with Biorendor.com. (**B**) Representative Western blot of mtDarT-3x-FLAG cells before (0 h) and after (4, 8 h) 100nM β-estradiol addition. Whole cell lysate is probed with Coomassie as loading control ($n = 3$ independent repeats). (**C**) Survival of yeast cells with empty vector or mtDarT plasmid, grown on YPG media with and without 100 nM β-estradiol. Representative image from three independent repeats is shown. Scale bar refers to the increasing dilution of cells from the left spot to the right-most spot. (**D**) Representative images of nucleoids stained with SGI and mitochondria marked with Cox4-mCherry. Dashed lines represent cell boundaries. (**E**) SGI staining in Δ*fzo1* cells (ρ^0, mtDNA absent). Dashed lines represent cell boundaries. Scale bar, 8 µm here, and in all other images. Source data are available online for this figure.

© The Author(s)

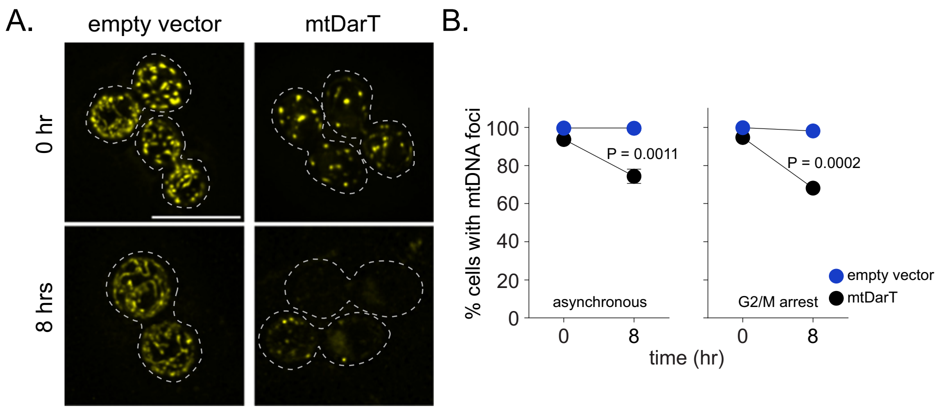

Figure EV2. mtDNA loss occurs independent of cell cycle progression.

(**A**) Representative images of nucleoid foci in G2/M-arrested, empty vector and mtDarT-expressing cells at 0 h (top) and 8 h after induction (bottom). Dashed lines represent cell boundaries. (**B**) Percentage of cells containing mtDNA foci in asynchronous (left) or G2/M-arrested (right) empty vector and mtDarT cells at 0 and 8 h after induction. Data shown from three independent repeats (n >138 cells per group, per repeat). Mean and SD are shown. Significance was calculated using Unpaired t test (two-tailed). **$P \leq 0.01$, ***$P \leq 0.001$. Scale bar, 8 µm here, and in all other images. Source data are available online for this figure.

© The Author(s)

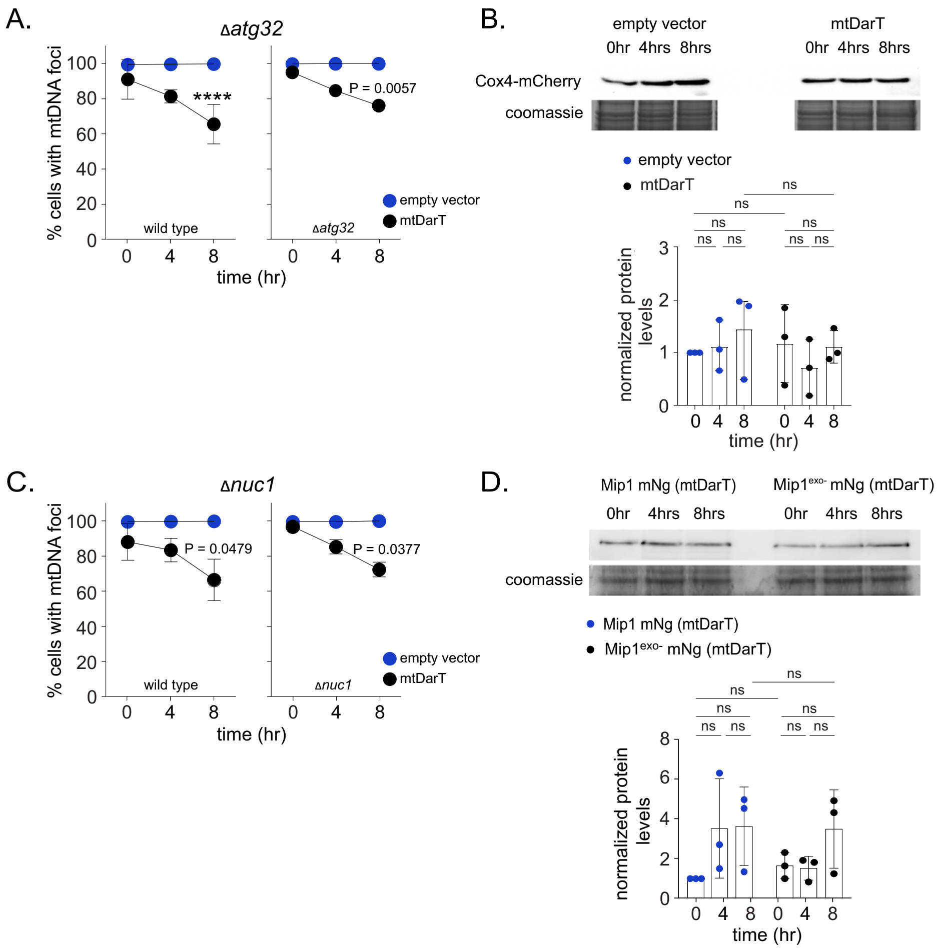

Figure EV3. mtDNA loss is dependent on Mip1 exonuclease.

(**A**) Percentage of cells with mtDNA foci in wild-type (left) or Δ*atg32* cells (right) at 0, 4 and 8 h after induction with empty vector (blue) and mtDarT (black). Data shown from three independent repeats (*n* >144 cells per group, per repeat). Mean and SD are shown. Significance was calculated using repeated measures one-way ANOVA and post hoc tests. $^{**}P \le 0.01$, $^{****}P \le 0.0001$. (**B**) Western blot of Cox4-mCherry in empty vector and mtDarT cells, before (0 h) and after (4, 8 h) damage induction. Representative western blot image is shown at the (top) and levels are quantified at the (bottom). $n = 3$ independent repeats. Mean and SD are shown. Significance was calculated using repeated measures one-way ANOVA and post hoc tests. (**C**) Percentage of cells with mtDNA foci in wild-type (left) or Δ*nuc1* cells (right) at 0, 4 and 8 h after induction with empty vector (blue) and mtDarT (black). Data shown from three independent repeats (*n* >115 cells per group, per repeat). Mean and SD are shown. Significance was calculated using repeated measures one-way ANOVA and post hoc tests. $^{*}P \le 0.05$. (**D**) Western blot of Mip1-mNeonGreen and Mip1^{exo-}-mNeonGreen before (0 h) and after (4, 8 h) damage induction. Representative western blot image is shown at the (top) and levels are quantified at the (bottom). $n = 3$ independent repeats. Mean and SD are shown. Significance was calculated using repeated measures one-way ANOVA and post hoc tests. Source data are available online for this figure.

 © The Author(s)

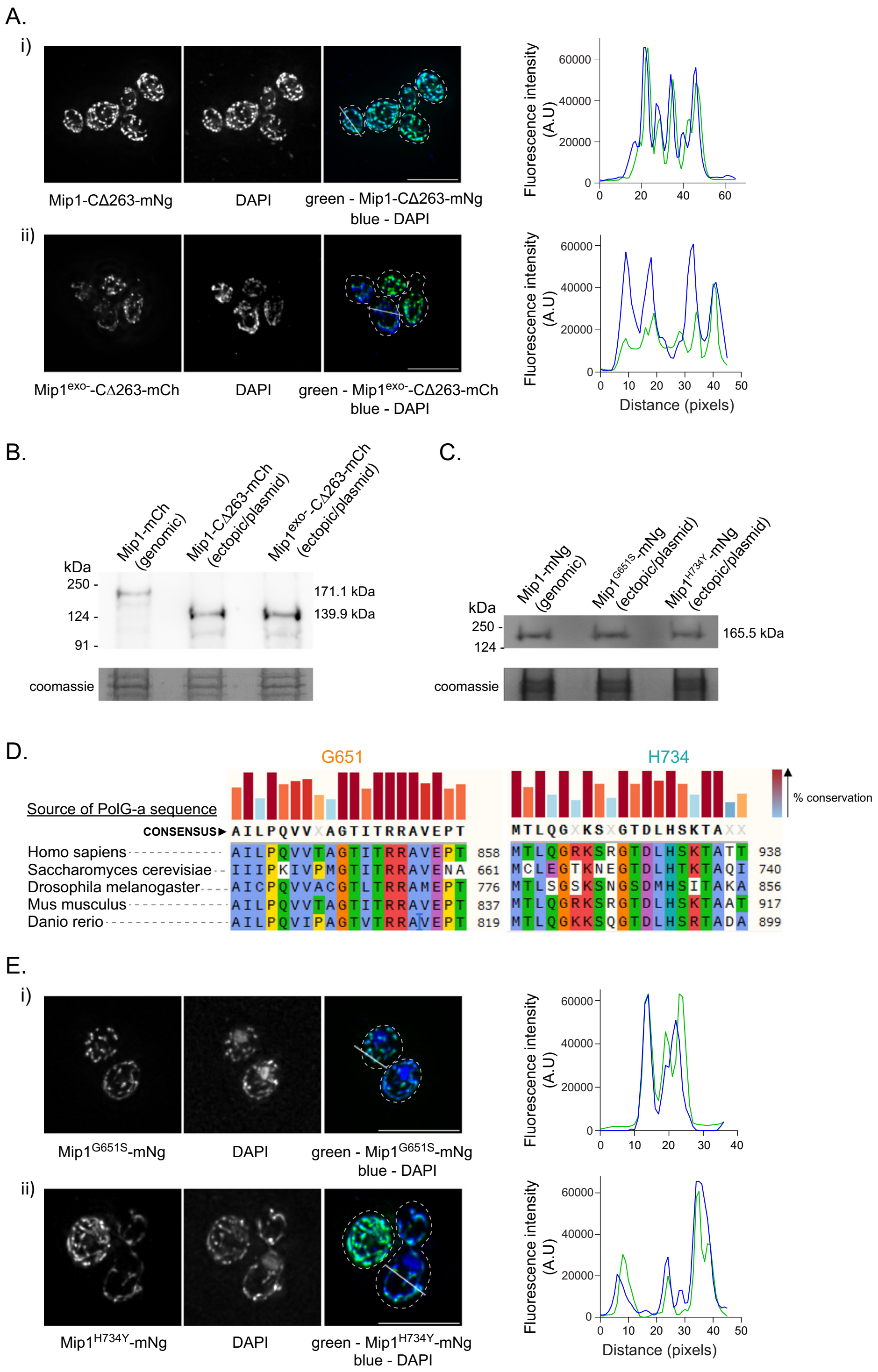
A.
i)
Mip1-CΔ263-mNg
DAPI
green - Mip1-CΔ263-mNg
blue - DAPI
ii)
Mip1exo--CΔ263-mCh
DAPI
green - Mip1exo--CΔ263-mCh
blue - DAPI
Fluorescence intensity (A.U)
Distance (pixels)
B.
Mip1-mCh (genomic)
Mip1-CΔ263-mCh (ectopic/plasmid)
Mip1exo- -CΔ263-mCh (ectopic/plasmid)
kDa
250 -
124 -
91 -
171.1 kDa
139.9 kDa
coomassie
C.
Mip1-mNg (genomic)
Mip1G651S-mNg (ectopic/plasmid)
Mip1H734Y -mNg (ectopic/plasmid)
kDa
250 -
124 -
165.5 kDa
coomassie
D.
G651
H734
% conservation
Source of PolG-a sequence
CONSENSUS▸ AILPQVVXAGTITRRAVEPT MTLQGXKSXGTDLHSKTAXX
Homo sapiens AILPQVVTAGTITRRAVEPT 858 MTLQGRKSRGTDLHSKTATT 938
Saccharomyces cerevisiae IIIPKIVPMGTITRRAVENA 661 MCLEGTKNEGTDLHTKTAQI 740
Drosophila melanogaster AICPQVVACGTLTRRAMEPT 776 MTLSGSKSNGSDMHSITAKA 856
Mus musculus AILPQVVTAGTITRRAVEPT 837 MTLQGRKSRGTDLHSKTAAT 917
Danio rerio AILPQVIPAGTVTRRAVEPT 819 MTLQGKKSQGTDLHSRTADA 899
E.
i)
Mip1G651S-mNg
DAPI
green - Mip1G651S-mNg
blue - DAPI
ii)
Mip1H734Y-mNg
DAPI
green - Mip1H734Y-mNg
blue - DAPI
Fluorescence intensity (A.U)
Distance (pixels)

Figure EV4. Exonuclease activity of Mip1 drives mtDNA loss under damage.

(**A**) Left: localization of Mip1-CΔ263-mNeonGreen (i) or *mip1*$^{exo-}$-CΔ263-mCherry (ii) and mt-nucleoids stained with DAPI in *mip1*$^{exo-}$ cells. Right: line profiles of Mip1-CΔ263-mNeonGreen (i) or Mip1^{exo-}-CΔ263-mCherry (ii) and mt-nucleoids stained with DAPI in *mip1*$^{exo-}$ cells. Line profile for a representative across an ROI is shown (white line in merged panel). Dashed lines represent cell boundaries. (**B**) Western blot of Mip1-mCherry, Mip1-CΔ263-mCherry and Mip1^{exo-}-CΔ263-mCherry. Representative image with loading control (Coomassie) is shown from one of the three independent repeats. (**C**) Western blot of Mip1-mNeonGreen, Mip1^{G651S}-mNeonGreen and Mip1^{H734Y}-mNeonGreen. Representative image with loading control (Coomassie) is shown from one of the three independent repeats. (**D**) Multiple sequence alignment of Human PolG and related orthologues, including Mip1, performed using COBALT to show the conservation and position of G651 and H734 across organisms. (**E**) Left: localization of Mip1^{G651S}-mNeonGreen (i) or Mip1^{H734Y}-mNeonGreen (ii) and mt-nucleoids stained with DAPI in *mip1*$^{exo-}$ cells. Right: line profiles of Mip1^{G651S}-mNeonGreen (i) or Mip1^{H734Y}-mNeonGreen (ii) and mt-nucleoids stained with DAPI in *mip1*$^{exo-}$ cells. Line profile for a representative across an ROI is shown (white line in merged panel). Dashed lines represent cell boundaries. Scale bar, 8 µm here, and in all other images. Source data are available online for this figure.

 © The Author(s)

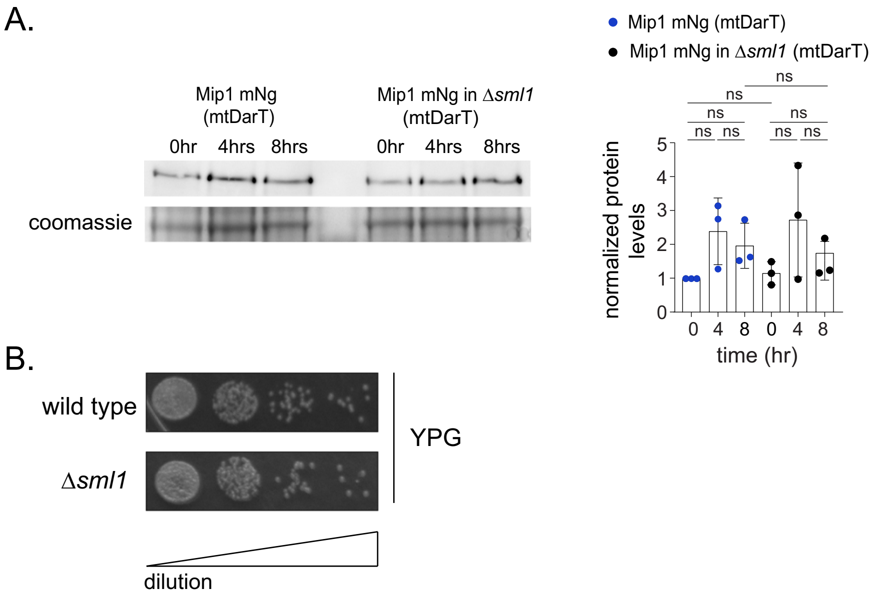

Figure EV5. Rates of mtDNA loss are influenced by dNTP levels.

(**A**) Western blot of Mip1-mNeonGreen in wild-type and Δ*sml1* cells before (0 h) and after (4, 8 h) damage induction. Representative western blot image is shown on the (left) and levels are quantified on the (right). $n = 3$ independent repeats. Mean and SD are shown. Significance was calculated using repeated measures one-way ANOVA and post hoc tests. (**B**) Serial dilution growth assay to measure growth of Δ*sml1* cells in comparison to a wild-type control. Representative image from three independent repeats is shown. Scale bar refers to the increasing dilution of cells from the left spot to the right-most spot. Source data are available online for this figure.
